# Analysis of macular blood flow changes in thyroid associated ophthalmopathy

**DOI:** 10.1186/s12886-022-02716-0

**Published:** 2022-12-20

**Authors:** Xiaohan Zhang, Wangyuan Liu, Zhaode Zhang, Jinhui Dai, Jinfeng Zhang, Lingli Lin

**Affiliations:** 1 Department of Ophthalmology, Ningde Municipal Hospital of Ningde Normal University, No.13 Mindong East Road, Fujian Ningde, China; 2 Department of Ophthalmology, Zhongshan Hospital, Fudan University, No.180 Fenglin Road, Shanghai, China

**Keywords:** Thyroid-associated ophthalmopathy, Vessel density, Foveal avascular zone, Optical coherence tomography angiography

## Abstract

**Background:**

To evaluate the changes in macular superficial retinal vessel density and their relation with visual acuity in thyroid-associated ophthalmopathy (TAO) patients with different severity.

**Methods:**

This cross-sectional observational study included 70 TAO patients and 70 healthy controls. Only data from the right eyes were analyzed. TAO patients were divided into 7 subgroups according to the NOSPECS score. Foveal avascular zone (FAZ), vascular density (VD), and perfusion density (PD) of macular 1 mm diameter and 6 mm diameter areas were measured by optical coherence tomography angiography (OCTA).

**Results:**

In TAO patients, significant increases were found in macular and foveal vascular densities (FVD) and perfusion densities (FPD) while a significant decrease was found in the FAZ area when compared with the control group (*p* < 0.05). Spearman correlation analysis and multiple linear regression analysis showed that TAO severity grade was negatively correlated with FVD (β = -1.150, *p* = 0.032), FPD (β = -0.024, *p* = 0.042), MVD (β = -0.583, *p* = 0.020) and MPD (β = -0.011, *p* = 0.010). Clinical activity score (CAS) score showed positive correlation with FVD (β = 0.794, *p* = 0.035) and FPD(β = 0.017, *p* = 0.041). FVD (β = -0.009, *p* = 0.033), MVD(β = -0.034, *p* < 0.001), FPD(β = -0.416, *p* = 0.039) and MPD(β = -2.428, *p* < 0.001) all showed negative correlation with best corrected visual acuity (BCVA).

**Conclusions:**

There was an overall increase in superficial macular blood flow in TAO patients compared with healthy controls and the blood flow decreased as TAO got worse. Superficial macular flow density was negatively correlated with BCVA.

## Background

Thyroid-associated ophthalmopathy (TAO) is an ocular manifestation of Graves’ Disease. Although the pathophysiology of TAO has yet to be fully revealed, it is now recognized as an autoimmune disorder and is closely related to the disfunction of systematic circulation [1–3]. Meanwhile, the retina microvasculature serves as a window for the observation of systemic microcirculation. A decrease in blood flow velocity of the superior ophthalmic vein and an increase in peak systolic velocity in the ophthalmic artery on color Doppler (CDFI) is closely associated with the severity of TAO [4], which could be used to evaluate hyperthyroid exophthalmia. Up to now, many techniques have been used to investigate the blood flow of retina and choroid in TAO, such as the Laser Doppler flow meter, indocyanine green angiography, and laser speckle imaging. However, the non-quantitative and invasive nature of these techniques set limitations for the observation of retinal microcirculation. As an important extension of optical coherence tomography (OCT), optical coherence tomography angiography (OCTA) is used to further observe the blood flow of retina and choroid. It shows good repeatability in quantitative analysis of retinal blood flow in normal eye-based optical microangiography (OMAG) [5]. Thus, as a novel non-invasive imaging study, OCTA has great potential for the evaluation of blood flow in the retina and choroid.

OMAG provides numerous quantitative parameters to analyze retinal blood flow, such as vessel density (VD) and perfusion density (PD). VD considers only the length of the blood flow signal which reflects the perfusion of the capillaries, while PD takes into account both the length and width of the blood flow signal. Recently, OCTA has been used to evaluate retinal blood flow, such as quantifying the macular vessel density in patients with diabetic retinopathy, branch vein occlusion, or glaucoma, and even been used to assess the impact of systemic diseases on retinal blood flow [6–8]. Ye [9] reported the use of OCTA to evaluate retinal microcirculation in active TAO patients, which showed changes in the macular microvasculature density in both the superficial and deep retina. However, this study did not compare the difference in retinal microcirculation in patients with different TAO severity.

In this study, we used OCTA to evaluate changes in superficial macular flow density and FAZ of ​​patients with different severity grades of TAO. Furthermore, we aim to explore the influence of TAO on superficial retinal microcirculation and the correlation between macular blood flow of ​​TAO and visual acuity.

## Methods

### Subjects

This cross-sectional observational study enrolled 70 healthy controls and 70 untreated TAO patients who were diagnosed in the Ophthalmology and Endocrinology departments in Ningde Hospital (Fujian, China) from June 2017 to June 2019. This study was approved by the Scientific and Ethical Committees of Ningde Hospital affiliated to Ningde Normal College and informed consent was obtained from all participants.

TAO was diagnosed according to the criteria proposed by Bartley and Gorman [10]. TAO patients were graded from 0 to 6 according to the NOSPECS classification [11] and 10 patients were enrolled in each grade*.* Grade 0: No symptoms and findings; Grade 1: No symptoms; only spasm in the upper eyelid, eye-opening increased; Grade 2: Swelling in the preorbital soft tissue; Grade 3: Proptosis; Grade 4: Involvement in extraocular muscle; Grade 5: Corneal involvement (only patients with corneal involvement that did not influence the OCTA signal were included in this study); Grade 6: Optic nerve involvement, visual loss in various levels. 70 healthy controls without systematic or ocular diseases were selected during their routine healthcare checkup in the Ophthalmology department (HC group). The healthy controls were matched with TAO patients in a similar age range(± 2 years). Best corrected visual acuity (BCVA), intraocular pressure, refraction, eyelid aperture, ocular protrusion, extra-ocular muscle thickness measured by B ultrasound, cornea evaluation, fundus examination, optic nerve OCT and clinical activity score (CAS) evaluation were evaluated in all participants [12]. The exclusion criteria for both TAO patients and healthy controls included: (1) any treatment for thyroid diseases, such as radioactive iodine therapy, immuno-suppressor agents, and thyroid surgery; (2) circulatory and metabolic diseases including diabetes, hypertension, and heart disease; (3) ocular diseases besides TAO that could potentially affect the retinal blood flow or visual acuity; (4) smoking and alcoholism; (5) long-term medication that could potentially affect the retinal blood flow or thyroid hormone; (6) Pregnant or breastfeeding; (7) History of ocular surgery. Only the right eyes were analyzed in this study.

### OCTA

**Fig. 1:**
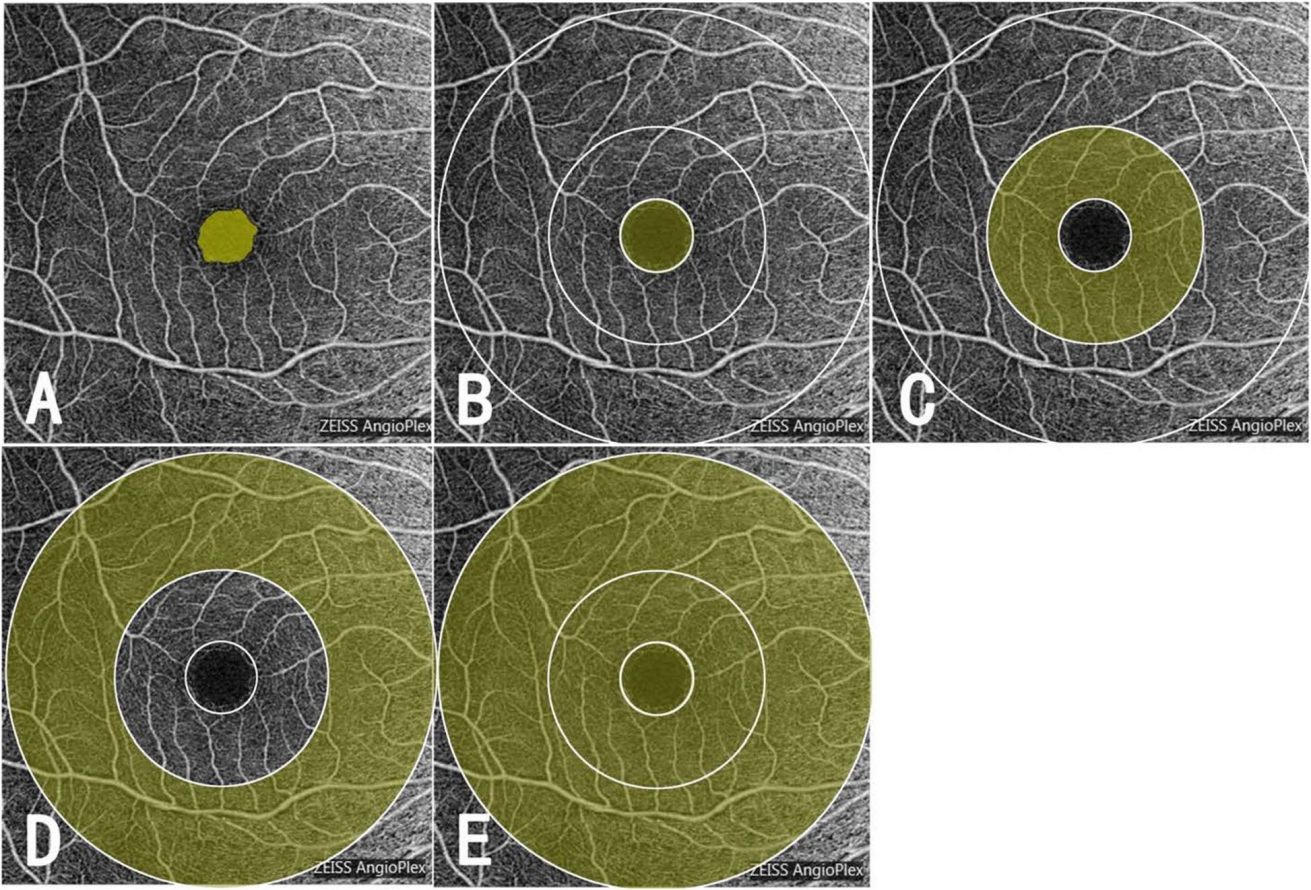
OCTA of the macular area (**A**) FAZ border after manual checking (**B**)-(**E**) ETDRS grid was divided into the following regions: region 1, central 1-mm (**B**) region 2, the inner ring (**C**) region 3, the outer ring (**D**) region 4, 6 mm circle (**E**). Macular flow density in region 1 was indicated by FVD and FPD; macular flow density in region 4 was represented by MVD and MPD

**Fig. 2:**
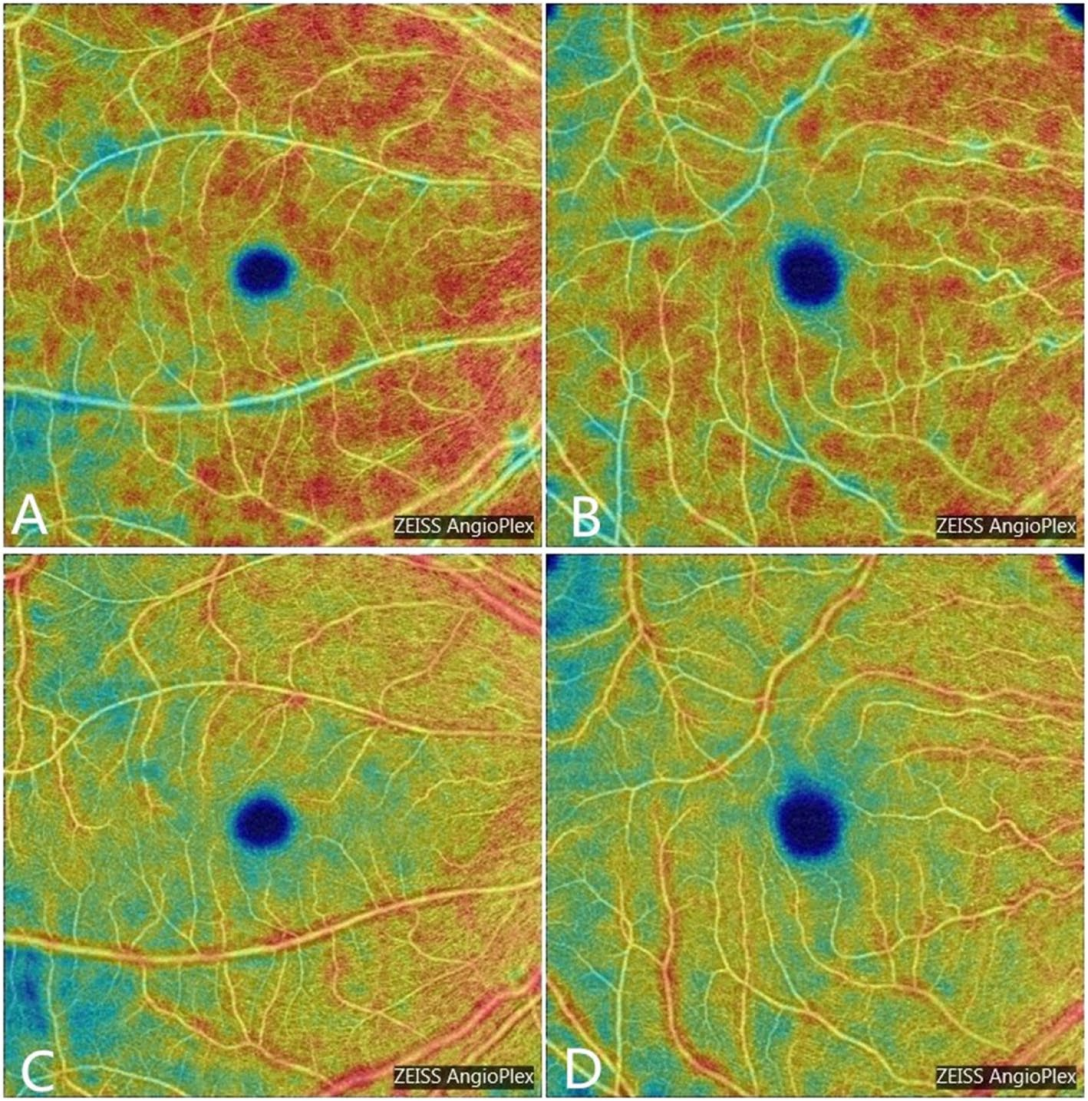
Pseudo-color images of macular (**A**) The pseudo-color images of MVD of a participant in the control group (**B**) The pseudo-color images of MVD of a patient with TAO (grade 1) (**C**) The pseudo-color images of MPD of a participant in the control group (**D**) The pseudo-color images of MPD of a patient with grade 1 TAO

OCTA was performed in all subjects. A Cirrus HD-OCT5000(Carl Zeiss, Germany) was used to scan the macular area with a scanning range of 6 mm × 6 mm. The subject was asked to place the chin and forehead on the chin rest and forehead rest respectively and stared at the green fixation cursor from the lens for more than 3 s for scanning. FastTrac@ image tracking function was turned on for all scans. OCTA image quality quantitative evaluation was adopted and images with a quality of < 8 were not included in the study. OCTA data was automatically imported into the FORUM system (version 4.0) and was quantitatively analyzed by its own analysis software (angio-analytics). The superficial retinal layer (SRL) was calculated from the internal limiting membrane (ILM) to the inner plexiform layer (IPL). A 6 × 6 mm macular scan was used to measure the superficial macular capillary plexus. The macular area was divided into the 1 mm central circle, 3 mm inner ring, 6 mm outer ring, and 6 mm circle according to the Early Treatment Diabetic Retinopathy Study (ETDRS) to quantitatively analyze PD and VD (Fig. 1). Because the 1 mm central circle and 6 mm circle could better represent the macular blood flow, we only analyzed these two regions in this study, which were the foveal vessel density (FVD) and foveal perfusion density (FPD) of the 1 mm diameter fovea and macular vessel density (MVD) and macular perfusion density (MPD) of the 6 mm diameter macula (Fig. 2). Vessel density was defined as the linear length of perfused retinal microvasculature per unit area in the region of measurement. Perfusion density was defined as the total area of perfused retinal microvasculature per unit area in a region of measurement. The FAZ boundaries were drawn automatically by the angiometric software from the manufacture and manually checked before the software automatically calculated the areas. The physicians for the manual check were masked to FAZ group allocation to avoid potential bias. All examinations were conducted by the same physician and interpreted by two independent physicians. A third physician would make the verdict should there be any disagreement.

### Statistical analysis

Because of the high correlation coefficients of parameters between left and right eyes, only the data from right eyes were analyzed. The normal distribution of data was assessed with the Shapiro–Wilk test. Normally distributed data were presented as means with standard deviations and non-normally distributed data were presented as median with quantiles. Chi-square analysis was used for categorical data. The influence of age, eyelid aperture, IOP, TAO grade, and CAS on FAZ and macular blood flow in the TAO group were analyzed using Spearman correlation test and multiple linear regression analysis. The effects of FAZ and retinal blood flow on BCVA in the TAO group were analyzed using linear regression. The differences in FAZ and retinal blood flow between subjects (control group and TAO subgroups) were analyzed using Kruskal–Wallis H test. The sample size was analyzed using the Power Analysis and Sample Size (PASS) (PASS 15.0.5, NCSS, LLC). Data analyses were performed using the Statistical Package for the Social Sciences (SPSS) (IBM SPSS Advanced Statistics 24.0, Armonk, NY, IBM Corp). Results were considered significant at the *P* < 0.05 level.

## Results

### Demographics and clinical characteristics

In total, 140 participants, comprising 70 TAO patients (70 eyes) and 70 healthy controls (70 eyes) were included in this study. The BCVA in the TAO group was significantly worse than the control group (*p* = 0.025). TAO group also showed higher palpebral fissures (14.00 mm, 14.00 mm ~ 15.00 mm, *p* < 0.001*). No significant difference was found in age, gender, and IOP between TAO and control groups (Table 1). In TAO subgroups, significant differences were found in IOP, CAS score, Palpebral fissure height, and BCVA (Table 2).

**Table 1:** Demographics and clinical characteristics of TAO and Control groups

	TAO (*n* = 70)	HC (*n* = 70)	*P*
Age (year)	38.00 (31.00,48.00)	42.00 (37.00,51.00)	0.269 *
Sex (male/female)	26/44	30/40	0.605†
IOP(mmHg)	16.00 (15.00,19.00)	14.00 (13.00,16.00)	0.124 *
BCVA (Log MAR)	0.00 (0.00,0.05)	0.00 (0.00,0.00)	0.259 *
Palpebral fissure height (mm)	14.00 (14.00,15.00)	12.00 (11.00,13.00)	< 0.001 *

*IOP* Intraocular pressure, *BCVA* Best-corrected visual acuity, variables were presented as median with quantiles

^*^Kruskal Wallis H test, †Chi-squared test

**Table 2:** Demographics and clinical characteristics of TAO subgroups

	TAO 0 (*n* = 10)	TAO 1 (*n* = 10)	TAO 2 (*n* = 10)	TAO 3 (*n* = 10)	TAO 4 (*n* = 10)	TAO 5 (*n* = 10)	TAO 6 (*n* = 10)	*P*
Age (year)	33.50 (30.00,39.25)	46.50 (31.00,50.50)	38.00 (28.5,46.75)	35.00 (32.75,48.50)	30.50 (23.00,48.00)	46.50 (45.00,48.00)	42.50 (33.50,50.25)	0.108*
IOP (mmHg)	14.00 (11.75,14.25)	15.00 (14,16)	16.00 (12.75,16.25)	16.50 (15.25,18.5)	17.00 (15.75,18.25)	19.00 (16.75,19.25)	20.5.00 (19.00,23.00)	< 0.001*
CAS	0.00 (0.00,0.00)	0.00 (0.00,1.00)	4.00 (3.00,4.00)	4.00 (2.50,5.00)	5.00 (3.50,5.00)	5.00 (3.50,5.00)	4.50 (3.00,6.00)	< 0.001*
Palpebral fissure height (mm)	14.00 (13.00,14.00)	14.00 (13.75,15.00)	14.00 (13.00,15.00)	14.00 (13.00,14.00)	14.00 (14.00,15.00)	15.50 (15.00,16.00)	18.00 (16.00,19.25)	< 0.001*
BCVA	0.00 (0.00,0.00)	0.00 (0.00,0.00)	0.00 (0.00,0.00)	0.00 (0.00,0.00)	0.00 (0.00,0.00)	0.00 (0.00,0.05)	0.26(0.15,0.52)	< 0.001*
Sex (male/female)	3\7	4\6	3\7	4\6	4\6	5\5	3\7	0.975†

*IOP* Intraocular pressure, *BCVA* Best-corrected visual acuity, *CAS* Clinical activity score, variables were presented as median with quantiles

^*^Kruskal Wallis H test, †Chi-squared test

### Differences in FAZ and macular blood flow between TAO and control groups

In the TAO group, FAZ significantly decreased compared with the control group while all the other macular blood flow parameters in TAO (FVD, MVD, FPD, and MPD) were significantly increased (Table 3).

**Table 3:** FAZ and macular blood flow differences between TAO and control groups

	FAZ (mm^2^)	FVD (mm^−1^)	MVD (mm^−1^)	FPD	MPD
TAO	0.22(0.14 ~ 0.30)	11.8(7.6 ~ 13.7)	18.5(18.0 ~ 19.0)	0.271(0.181 ~ 0.314)	0.459(0.446 ~ 0.466)
HC	0.34(0.29 ~ 0.43)	7.3(4.9 ~ 9.0)	17.7(17.3 ~ 18.2)	0.164(0.109 ~ 0.192)	0.439(0.426 ~ 0.452)
Z	-6.446	-5.705	-4.617	-6.124	-4.597
p	< 0.001	< 0.001	< 0.001	< 0.001	< 0.001

*FAZ* foveal avascular zone, *FVD* foveal vessel density, *MVD* macular vessel density, *FPD* foveal perfusion density, *MPD* macular perfusion density, F degrees of freedom in Kruskal–Wallis H test (control group and 7 TAO subgroups)

### Differences in FAZ, BCVA, and macular blood flow between TAO subgroups and control group

**Fig. 3:**
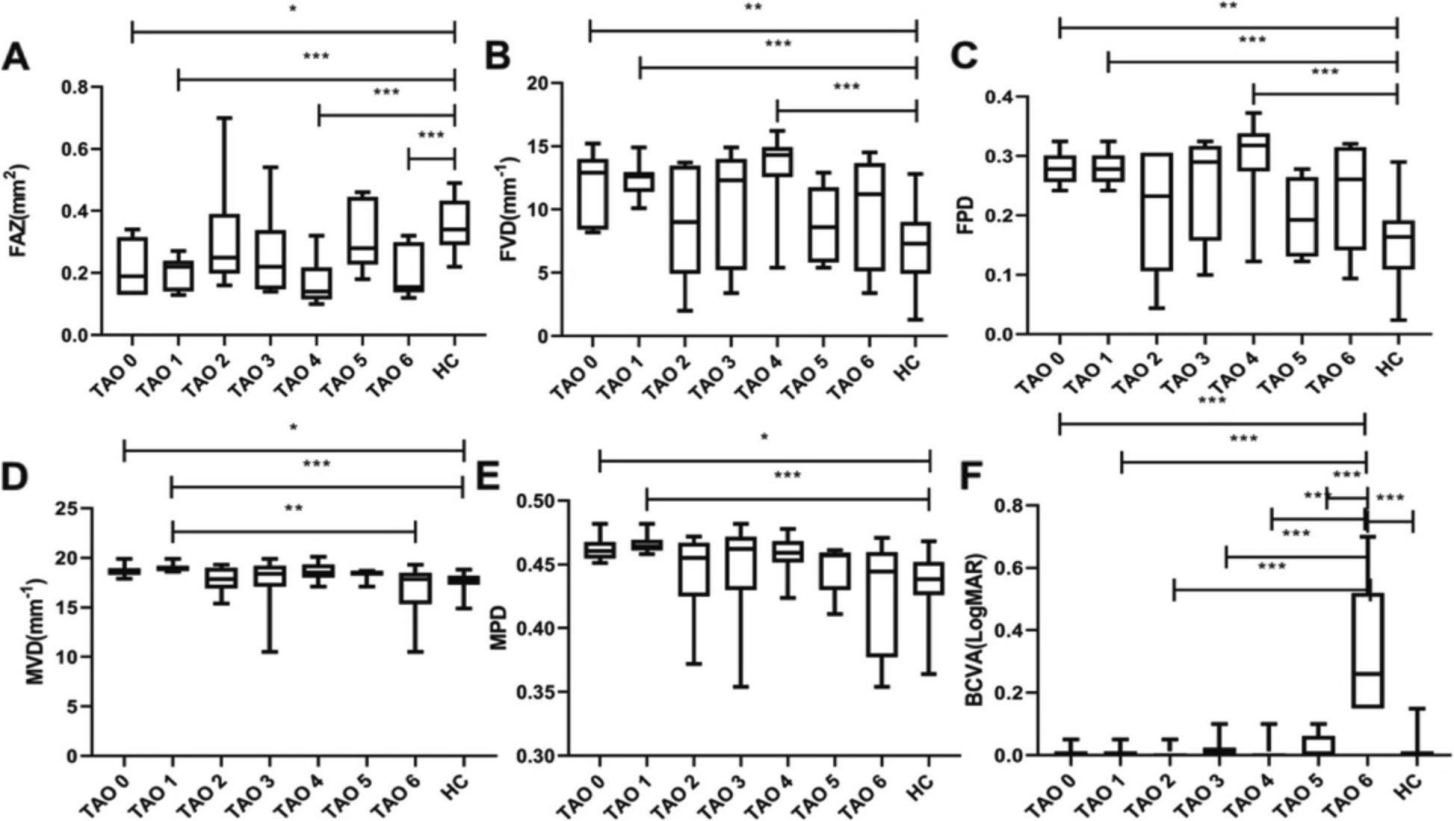
Intra-group comparisons in FAZ, BCVA, and macular blood flow among TAO subgroups and control group (**A**) Intra-group comparison in FAZ (**B**) Intra-group comparison in FVD (**C**) Intra-group comparison in FPD (**D**) Intra-group comparison in MVD (**E**) Intra-group comparison in FAZ (**F**) Intra-group comparison in BCVA. FAZ foveal avascular zone, FVD foveal vessel density, MVD macular vessel density, FPD foveal perfusion density, MPD macular perfusion density, BCVA best corrected visual acuity. * *p* < 0.05; **p < 0.01; *** *p* < 0.001

Kruskal–Wallis H test showed that there were significant differences among 7 TAO subgroups and the control group in FAZ, FVD, MVD, FPD, MPD, and BCVA. Further intra-group analysis showed that significant decrease was found in FAZ in TAO grade0, grade1, grade4 and grade6 comparing to control group (*p* = 0.011, < 0.001, < 0.001, < 0.001). Moreover, significant increase was found in FVD and FPD in TAO grade0, grade1, and grade4 comparing to control group (p = 0.005, 0.001, < 0.001; *p* = 0.003,0.001, < 0.001), while only TAO grade0 and grade1 showed increase in MVD and MPD comparing to control group (*p* = 0.016, < 0.001; *p* = 0.011, < 0.001). TAO grade6 showed a significant decrease in MVD compared to TAO1(*p* = 0.007) as well as the worst BCVA than all the other 7 groups (all *p* < 0.001) (Fig. 3).

### Correlation between ocular parameters and macular blood flow

Age, palpebral fissure height, IOP, TAO severity grade, and CAS score were included in Spearman correlation analysis and multiple linear regression analysis (Table 4). The analysis showed that TAO severity grade, CAS score, age, palpebral fissure height, and IOP were not influencing factors of FAZ in TAO patients. TAO classification and age were negatively correlated with FVD and FPD in TAO patients, while CAS score was positively correlated with FVD and FPD in TAO patients. The palpebral fissure height and IOP were not independent influencing factors of FVD and FPD in TAO patients. TAO classification was negatively correlated with MVD and MPD in TAO patients. CAS score, age, eyelid aperture, and IOP were not independent influencing factors of MVD and MPD in TAO patients.

**Table 4:** Correlation between ocular parameters and macular blood flow

**Dependent variable**	**TAO grade**			**CAS**			**Age(year)**			**IOP(mmHg)**			**Palpebral fissure height(mm)**			**F**	**P**	**R**^**2**^
	**B**	**Beta**	***P*-value**	**B**	**Beta**	***P*-value**	**B**	**Beta**	***P*-value**	**B**	**Beta**	***P*-value**	**B**	**Beta**	***P*-value**			
**FAZ(mm**^**2**^**)**	0.016	0.246	0.437	-0.009	-0.156	0.506	0.003	0.210	0.138	-0.006	-0.135	0.481	-0.004	-0.058	0.740	1.172	0.333	0.084
**FVD (mm**^**−1**^**)**	-1.150	-0.618	**0.032**	0.794	0.449	**0.035**	-0.114	-0.300	**0.019**	0.147	0.118	0.492	0.239	0.106	0.494	4.588	**0.001**	0.264
**MVD (mm**^**−**^**)**	-0.583	-0.695	**0.020**	0.340	0.426	0.053	-0.033	-0.191	0.146	0.059	0.105	0.555	0.088	0.087	0.588	3.434	**0.008**	0.212
**FPD**	-0.024	-0.599	**0.042**	0.017	0.445	**0.041**	-0.002	-0.259	**0.048**	0.003	0.124	0.482	0.003	0.072	0.649	3.759	**0.005**	0.227
**MPD**	-0.011	-0.748	**0.010**	0.006	0.413	0.052	-0.010	-0.207	0.103	0.001	0.054	0.753	0.002	0.111	0.477	4.611	**0.001**	0.265

*FAZ* foveal avascular zone, *FVD* foveal vessel density, *MVD* macular vessel density, *FPD* foveal perfusion density, *MPD* macular perfusion density, *B* Non-standardized coefficient, *Beta* B-standardized coefficient

Boldface numbers indicate statistically significant differences at *p* < 0.05

### Correlation between macular blood flow and BCVA in TAO patients

A negative correlation was found between BCVA (log MAR) and FVD, MVD, FPD, and MPD, which indicated that the BCVA decreased with the macular flow density decreasing in 1 mm diameter and 6 mm diameter macular areas. No significant correlation was found between BCVA and FAZ (Table 5).

**Table 5:** Influence of FAZ and macular blood blow on BCVA(LogMAR) in TAO patients

	**B**	**Std.Error**	**Beta**	**t**	***P***	***R***^**2**^
**FAZ**	-0.035	0.129	-0.033	-0.272	0.786	0.001
**FVD**	-0.009	0.004	-0.255	-2.176	**0.033**	0.065
**MVD**	-0.034	0.009	-0.425	-3.874	** < 0.001**	0.181
**FPD**	-0.416	0.198	-0.247	-2.103	**0.039**	0.061
**MPD**	-2.428	0.495	-0.511	-4.907	** < 0.001**	0.261
**Dependent variable: BCVA(LogMAR)**						

*FAZ* foveal avascular zone, *FVD* foveal vessel density, *MVD* macular vessel density, *FPD* foveal perfusion density, *MPD* macular perfusion density, *B* Non-standardized coefficient, *Beta* B-standardized coefficient

Boldface numbers indicate statistically significant differences at *p* < 0.05

## Discussion

TAO is one of the complications of Graves’s Disease that mainly caused by the inflammation and tissue expansion of the orbit. Efforts had been made to quantify TAO disease activity. OCTA offers a fast, convenient, non-invasive, and easy approach to quantitatively measure the blood flow in the macular area, and it also provides a way to evaluate and predict the severity of TAO. In this study, we used OCTA to measure the macular blood flow and FAZ area in TAO patients. Compared with the control group, the TAO group showed a significant decrease in FAZ area and an increase in FVD, FPD, MVD, and MPD. In order to further explore the relationship between the severity of TAO and the macular blood flow, we further divided the TAO group into 7 subgroups based on the NOSPECS score and investigated the changes in retinal microcirculation and FAZ area.

Previous studies on TAO macular blood flows using OCTA showed that superficial vessel density was lower in active TAO [13], while the superficial vessel density in inactive TAO varied in different studies. Yu [14]and Cetin [15] found a significantly higher retinal vascular density in inactive TAO, while Frazil [16] and Dave [17] found retinal vessel density values were similar in inactive TAO to healthy subjects. Moreover, Natasha [18] observed a decrease in macular VD in inactive TAO patients. This difference might be caused by the different medications that inactive TAO patients had received for thyroid disease. Therefore, in our study, only patients without any treatment for thyroid disease were included. We observed an overall increase in superficial vessel density in TAO patients compared to controls. We further classified TAO patients based on the NOSPECS score. Our analysis showed that as the severity of TAO increased, the 1 mm and 6 mm diameter macular vessel densities decreased. Further subgroup analysis also revealed that compared to the control group, only TAO grade0 and grade1 showed a significant increase in superficial vessel density. Our results suggested that macular flow density increased significantly in the early stage of the disease and then gradually decreased and stabilized.

Both circulation and ocular factors contribute to changes in blood flow in TAO. Systemic blood pressure and cardiac output were elevated in hyperthyroidism which may lead to an increase in orbital blood flow [19]. Mehmet [20] also observed an increase in systolic central retinal blood flow velocity in TAO patients using color Doppler ultrasound. Moreover, Kurioka [21] found that the orbital inflammatory changes in TAO also caused an increase in blood flow velocity in most TAO patients. However, in the late stage of TAO, degeneration and fibrosis of orbital muscles and adipose tissues lead to a decrease in retinal flow density.

In our study, an increase in macular flow density was observed in TAO grade 0 and grade 1. This might be caused by an increase in systemic blood pressure and cardiac output as a result of hyperthyroidism rather than ocular factors since minor TAO-related ocular changes were observed in TAO grade 0 and grade 1. With the progression of TAO, ocular factors took the helm and a trend of decrease in macular blood flow was observed in TAO subgroups.

It should be noted that TAO grade 4 showed an increase in foveal vessel density compared with the control group while no significant increase was found in macular vessel density. A previous study by Perri [22] reported that extraocular muscle hypertrophy was positively correlated with retinal blood flow. Thus, we speculated that the transient increase in foveal vessel density in TAO grade 4 was the combined effects of extraocular muscle hypertrophy and disease activity. The microcirculation of the patient's macular area is related not only to the severity of the disease, but also to the local inflammation and extraocular muscles.

Previous research found an enlargement of the FAZ area in active TAO while no significant change in non-active TAO [13]. In our study, we found an overall decrease in FAZ area in TAO patients while further analysis showed no correlation between the severity of TAO disease and FAZ area. After subgroup analysis, the FAZ areas of TAO grade0, 1, 4, and 6 were significantly smaller than that of the control group. This result is consistent with the increase of foveal vessel density in TAO grade0, 1, 4, and 6 compared with the control group, where the central avascular area narrows as the foveal vessel density enlarges. In addition, our analysis showed no significant correlation between the FAZ area and BCVA. This finding suggested that FAZ area changing is not the cause of BCVA decline in TAO, which is different from the pathological mechanism of diabetic retinopathy, retinal vein occlusion and other diseases that destroyed arch ring caused ischemia of the foveal and decrease in visual acuity. However, our analysis showed that macular vessel density positively correlated with BCVA, which suggested that the TAO-caused BCVA decline might be caused by macular microcirculation change. Therefore, we hypothesized that the changes in macular microcirculation have already affected visual function before the massive decline in BCVA in TAO grade 6 caused by optic nerve damage. The macular vessel density especially foveal vessel density showed a decrease in TAO grade 5 while BCVA(LogMAR) started to increase, which indicated the macular microcirculation change in TAO grade 5 has already had an impact on visual function. We speculated that the abundant blood flow and fast metabolic rate of the macular area in TAO patients also made it sensitive to hypoxia. Thus, the slight decline in flow density might influence the visual function in TAO patients. The BCVA(LogMAR) of TAO grade 6 increased significantly while the macular flow density in TAO grade 6 was not significantly decreased compared with in TAO grade 5. Thus, we considered that optic nerve damage, which is the main characteristic of entering TAO grade 6, played a decisive role in visual acuity decline in TAO grade 6. In summary, we believe that BCVA of middle and late stages TAO patients is also affected by changes in the macular microcirculation. When the TAO severity is assessed as grade 4, the macular flow density, especially FVD and FPD, should be monitored regularly. Once a significant decline in macular flow density is detected, it is necessary to be alerted that TAO would progress into grade 5. At this stage, besides conventional treatment, additional clinical intervention to promote retinal microcirculation and protect visual function should be considered.

The major limitation of this study is the lack of large sample sizes in each subgroup. Further cross-sectional observational studies and longitudinal follow-up studies with larger sample sizes are needed to dynamically observe the changes in retinal blood flow in TAO. Our study is the first horizontal study on macular OCTA parameters in TAO patients classified by NOSPECS score. Moreover, all the TAO patients included in this study were initially diagnosed without any medical interventions, which excluded the influences of previous TAO severity and medical treatment on the macular blood vessels. Because shadows and projection artifacts in OCTA have a greater impact on deep retinal blood flow, only superficial retinal blood flow parameters were analyzed. Therefore, the blood flow changes in the deep retina of TAO patients need to be further explored.

## Conclusions

Our study analyzed the superficial macular flow density and FAZ area in TAO patients with different severity. Our result showed that overall superficial vessel density increased in TAO patients compared with healthy controls and there was a trend of decrease as TAO got more severe. FAZ area showed an increase in TAO patients but this change was not correlated with TAO severity. Our study suggested that superficial macular flow density was negatively correlated with BCVA(LogMAR). Thus, when TAO NOSPECS grade reaches grade 5, systemic and ocular microcirculation intervention should be considered to protect visual function.

## Data Availability

The datasets used and/ analyzed during the current study are available from the corresponding author on reasonable request.

## Abbreviations

TAO: Thyroid-associated ophthalmopathy
FAZ: Foveal avascular zone
VD: Vascular density
PD: Perfusion density
OCTA: Optical coherence tomography angiography
FVD: Foveal vascular densities
FPD: Foveal perfusion densities
CAS: Clinical activity score
BCVA: Best corrected visual acuity
OCT: Optical coherence tomography
OMAG: Normal eye-based optical microangiography
SRL: Superficial retinal layer
ILM: Internal limiting membrane
IPL: Inner plexiform layer
EDTRS: Early Treatment Diabetic Retinopathy Study
CT: Computed Tomography
MRI: Magnetic Resonance Imaging
